# A Case Report of Basilar Artery Occlusion in a Healthy 36-Year-Old Female

**DOI:** 10.7759/cureus.8904

**Published:** 2020-06-29

**Authors:** Carl E Rhodes, Thomas Kelleher, Cherian I Plamoottil

**Affiliations:** 1 Emergency Medicine, Midwestern University Chicago College of Osteopathic Medicine, Chicago, USA; 2 Neurology, Midwestern University Chicago College of Osteopathic Medicine, Chicago, USA; 3 Emergency Medicine, Osceola Regional Medical Center, Orlando, USA

**Keywords:** stroke, basilar artery, posterior circulation

## Abstract

Basilar artery occlusion is rare, accounting for approximately 1% of strokes. Symptoms range from paresthesia and oculomotor symptoms to locked-in syndrome. Intervention can lead to complete neurological recovery despite the severity of initial deficits. We report a case of basilar artery occlusion in a healthy 36-year-old female with minimal risk factors. The patient underwent interventional thrombectomy within eight hours of onset of symptoms and made a significant recovery. Due to the variation in severity and character of symptoms, the diagnosis of basilar artery occlusion is often a barrier to care.

## Introduction

Basilar artery occlusion is rare, accounting for approximately 1% of strokes [1]. The basilar artery is a major component of the posterior circulation, contributing to the circle of Willis and supplying the structures of the posterior cranial fossa including the pons and cerebellum. It runs anterior to the brainstem and is formed by the union of the vertebral arteries. Occlusion of the basilar artery can have a variety of clinical manifestations ranging from transient weakness or paresthesia to near-complete paralysis. Complete occlusion of the proximal or middle basilar artery leads to ischemia of the paramedian base of the pons but spares the tegmentum. The result is locked-in syndrome, in which consciousness and oculomotor function are preserved, but all other voluntary muscle movement is lost. A complete distal basilar artery occlusion (the “top of the basilar” syndrome) can cause ischemia to the midbrain and thalamus, most often resulting in oculomotor abnormalities and alterations in alertness and behavior. Partial occlusions of the basilar artery can lead to a variety of deficits depending on the location and severity of the occlusion, and the anatomical regions affected.

The symptoms associated with basilar artery occlusion can be abrupt, particularly in embolic and distal occlusions, but many patients with atherosclerotic and proximal occlusions experience prodromal symptoms. Patients can experience a near-complete recovery if treatment is provided promptly. However, the time from onset of symptoms to diagnosis in the emergency department is often significantly delayed in basilar artery occlusion, with one study reporting an average total delay of 16 hours and eight minutes [2]. Treatment of basilar artery occlusion involves local intra-arterial thrombolysis (IAT) where possible, or intravenous thrombolysis (IVT). Lindsberg and Mattle found that recanalization is frequently achieved in both IAT (225 of 344; 65%) and IVT (40 of 76; 53%). Despite this, survival rates after IVT (38 of 76; 50%) and IAT (154 of 344; 45%) were equal (P=0.48) [3].

## Case presentation

**Figure 1:**
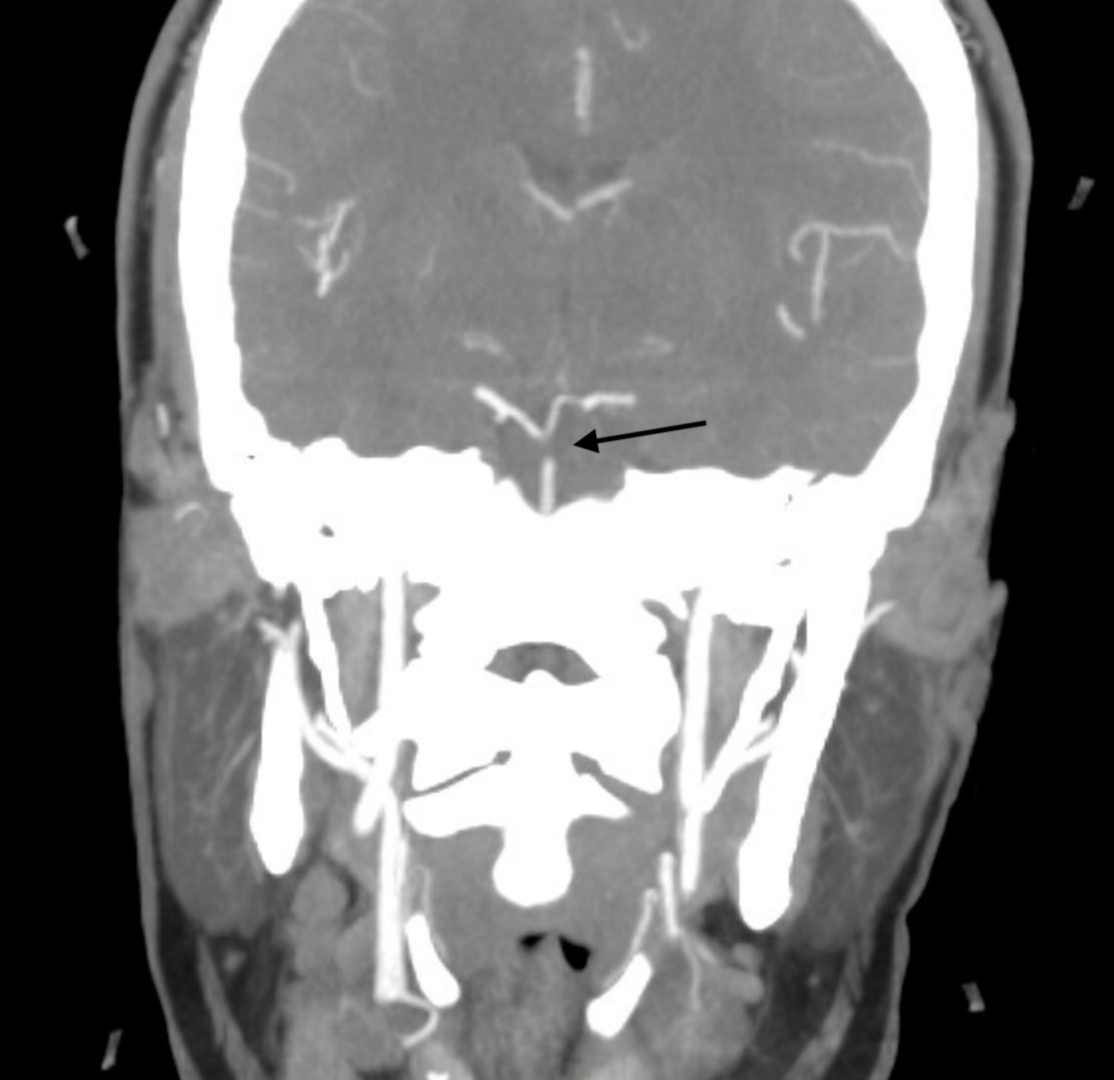
Filling defect in the distal aspect of the basilar artery (black arrow), suspicious for thrombus.

Our patient is a 36-year-old female with no past medical history. She has never smoked cigarettes and does not consume alcohol. She denied any history of clots, prior miscarriages, or family history of stroke. Her risk factors for stroke are oral contraceptive use, morbid obesity, and the recent use of weight loss supplements. Our patient awoke at approximately 06:30 with symptoms of dizziness, vomiting, head and neck pain, and left lip paresthesia. Her last known normal was 22:30 the previous night. She presented to the emergency department at 08:30, where a focused physical exam also revealed left eye ptosis, right medial gaze palsy, and right upper extremity dysmetria. A non-contrast head CT revealed no acute intracranial abnormality. Follow-up CT angiogram of the head and neck revealed a filling defect in the distal aspect of the basilar artery, suspicious for a thrombus (Figure 1). Upon re-examination of the prior non-contrast CT, a subtle focus of increased density was identified anterior to the pons, representing a possible thrombus. At 13:00, the patient was noted to have developed mild right-sided facial droop. The patient was transferred to a tertiary care center for emergent endovascular thrombectomy at 14:00. Following successful thrombolysis in cerebral infarction (TICI) grade 3 thrombectomy, the patient experienced a significant improvement of her symptoms, with only mild right facial droop and right internuclear ophthalmoplegia noted on physical exam. MRI was remarkable for acute infarcts of the right midbrain, right pons, and left cerebellar hemisphere.

Once the patient was stabilized, a transesophageal echocardiogram (TEE) was ordered, and further history was elicited to evaluate the cause of the patient’s stroke. TEE with agitated saline contrast revealed no evidence of shunt. She was discharged to an acute rehab facility with a 30-day cardiac monitor and instructed to take aspirin 325 mg and atorvastatin 80 mg daily.

## Discussion

Basilar artery thrombosis in a healthy 36-year-old female with successful IVT is a rare occurrence. According to a query of the Healthcare Cost and Utilization Project (HCUP) National Emergency Department Sample for 2016, there were 123 (±27) emergency department visits nationwide carrying a diagnosis of cerebral infarction due to thrombosis of the basilar artery. Death or dependency was seen in 76% (260/344) receiving IAT, despite recanalization occurring in 65% of patients [3].

Although the patient is young and does not have any medical diagnosis that would increase her risk of stroke, she does have many potential risk factors. These include morbid obesity, possible undiagnosed diabetes mellitus, use of oral contraceptives, and use of unknown weight loss supplements. The patient is morbidly obese with a body mass index of 41.8 kg/m^2^. In a population-based case-control study, there was noted to be a correlation between obesity and an increased likelihood of ischemic stroke with an odds ratio of 1.57 (1.28-1.94) [4]. The mechanism behind this association is unclear but may be due to comorbid conditions such as diabetes mellitus and hypertension. The patient did have elevated blood pressure upon admission and a glucose of 176. Given her elevated glucose, it is possible she has undiagnosed type 2 diabetes mellitus. The Emerging Risk Factors Collaboration study showed an adjusted hazard ratio of 2.27 (1.95-2.65) in diabetic patients for ischemic stroke [5]. The mechanism behind this association is likely due to increases in systemic inflammation and arterial stiffness, which increase the likelihood of atherosclerosis. Diabetes also increases the risk of hypertension, microvascular disease, lipid abnormalities, and hyperglycemia, which can increase the likelihood of congestive heart disease and subsequent stroke. Finally, a 2015 meta-analysis of the risks associated with combined oral contraceptives demonstrated a relative risk of arterial thrombosis of 1.6 when compared to non-users (95% CI 1.3-1.9) [6].

Although the patient is obese and uses oral contraceptives, the development of a basilar artery occlusion is still highly unusual at such a young age. This could indicate another contributing factor at play. The patient began taking weight loss supplementation a few weeks before onset of symptoms. Supplements are not regulated by the Food and Drug Administration and may cause a variety of medical illnesses. Specifically, the components of the supplement may not be safe and may not be in the quantity that is listed. Weight loss supplements, in particular, have been shown to be dangerous. In a case study published in Hindawi journal, the weight loss supplement 3,3'-diindolylmethane was shown to have potentially caused a pulmonary embolism and deep vein thrombosis in a 65-year-old male [7].

Along with estrogen-like compounds, weight loss supplements often have stimulants. Ephedrine, a potent stimulant, was used as a weight loss substance for many years before being banned because of an association with sudden death. A related compound, synephrine, is still being used. These substances can cause atrial fibrillation which can increase the risk of stroke [8]. A mural thrombus can develop in the atria due to incomplete atrial contraction. A clot can then dislodge and make its way into the systemic circulation causing an ischemic stroke. Though atrial fibrillation was not captured on electrocardiogram, the patient did have a prolonged QTc interval. Recent research has shown that prolonged QTc can predispose patients to developing atrial fibrillation [9]. Furthermore, when controlling for comorbidities such as hypertension and atrial fibrillation, QTc is associated with an increased risk of ischemic stroke. This suggests that QTc could be an early marker of atherosclerotic disease [10].

## Conclusions

We describe a 36-year-old female with no past medical history who suffered a thrombotic basilar artery occlusion despite minimal risk factors. Patients with basilar artery occlusion can experience significant recovery following reperfusion therapy, but delay in presentation to the emergency department and diagnosis of basilar artery occlusion is a barrier to care. The symptoms of basilar artery occlusion are varied in both severity and character, possibly contributing to the delay in diagnosis and treatment.
